# Impact before and after introduction of a multifaceted quality improvement intervention on device-related infections in a pediatric ICU in India: a single-centre experience

**DOI:** 10.1186/cc14052

**Published:** 2014-12-03

**Authors:** R Rameshkumar, A Jagadeesh, M Kedarnath, S Mahadevan, P Narayanan, KN Harikrishnan, S Sistla

**Affiliations:** 1 Department of Pediatrics, Jawaharlal Institute of Postgraduate Medical Education & Research, Puducherry, India; 2 Department of Microbiology, Jawaharlal Institute of Postgraduate Medical Education & Research, Puducherry, India

## Introduction

Healthcare-associated infections (HAI) are a significant problem in the pediatric intensive care unit (PICU). Apart from contributing to mortality, they also increase the coast of PICU care [1]. Surveillance and prevention of HAI by multifaceted quality improvement intervention among critically ill children remain part of the standard of care [2].

## Methods

The study was conducted in the 19-bed PICU of a tertiary care referral academic institute. Data regarding ventilator-associated pneumonia (VAP) and central line associated bloodstream infection (CLABSI) using the CDC definition were collected prospectively from July 2013 to June 2014. Multifaceted quality improvement intervention consisting of infection control nurse and physician and hand hygiene education module and wearing a gown and mask during the care of critically ill children was introduced from January 2014. Incidence of VAP and CLABSI was compared before (July to December 2013) and after (January to June 2014) introduction of the intervention.

## Results

Before the intervention period, the incidence of VAP was 28.5 per 1,000 ventilation-days and CLABSI was 13.7 per 1,000 catheter-days. After the intervention period the incidence of VAP was 13.3 per 1,000 ventilation-days and CLABSI was 8.3 per 1,000 catheter-days. The proportion of patients ventilated for more than 48 hours who had VAP was significantly less after intervention as compared to before the intervention period (14.2%, *n *= 25/176 vs. 25.2%, *n *= 29/155; *P *= 0.012, odds ratio (OR), 95% CI 0.49, 0.28 to 0.86). Both groups were similar with respect to age, sex ratio, severity (PRISM-III), device utilization rate and grade of infection. No significant difference occurred in overall PICU mortality before and after intervention (28.2% vs. 28.9%).

## Conclusion

Multifaceted quality improvement intervention results in significant reduction of the healthcare-associated infection rate although it was higher than reported from developed countries [1-3].
